# Notes for authors 2012

**DOI:** 10.1107/S1600536811047003

**Published:** 2011-12-21

**Authors:** IUCr Editorial Office

**Affiliations:** a 5 Abbey Square, Chester CH1 2HU, England

**Keywords:** Notes for authors

## Abstract

Notes for authors.

*Acta Crystallographica Section E: Structure Reports Online* is the IUCr’s highly popular electronic-only structural journal. It provides a fast, simple and easily accessible publication mechanism for the growing number of valuable crystal structure determinations of inorganic, metal-organic and organic compounds. The electronic submission, validation, refereeing and publication facilities of the journal ensure rapid and high-quality publication, whilst key indicator flags are used to provide measures of structural reliability. The journal encourages the publication of routine as well as interesting and difficult structures.

Articles are published in a short-format style with enhanced supplementary materials (see §8.1 for details of an example submission and publication). Each publication consists of a complete package – the published article, HTML and PDF supplements, CIF, structure factors, graphics, and any other submitted supplementary files. This represents a much richer collection of material than is provided by publishers of other structural journals.

*Acta Crystallographica Section E* is an open-access journal, *i.e.* free of charge to all readers. The costs of peer review, of journal production, and of online hosting and archiving will be met by charging an open-access fee to authors (see §9).

## Submission requirements

### Manuscript checking and preparation

All articles must be submitted in Crystallographic Information File (CIF) format. Details about CIFs are given in Hall *et al*. [*Acta Cryst*. (1991), A**47**, 655–685]. Guidelines for the preparation and editing of a CIF, the data items required therein, standard data codes and keywords, CIF templates, example CIFs, and data-validation criteria and procedures are available *via* the online author help page (see §8). A CIF editor for authors, *publCIF*, may be obtained from **http://publcif.iucr.org**, and a general CIF editor (*enCIFer*) may be obtained from **http://www.ccdc.cam.ac.uk/products/encifer/index.html**; both editors are available free of charge. The Section Editors, Co-editors and Editorial Office staff are also available to assist any author with any technical CIF problems. Please note that this help does not include rewriting articles which do not reach the standards specified in §1.10.

Before submission, authors are required to check their CIF and associated structure factors using the *checkCIF* service at **http://journals.iucr.org/services/cif/checking/checkfull.html**. All validation alerts returned by checkcif should be considered carefully and corrected as far as possible. Sometimes these alerts relate to simple omissions or errors in the CIF. If the report contains validation alerts about the consistency, adequacy or quality of the data or the refined model, these will need to be addressed, or, if the authors consider there are specific valid or unavoidable reasons for these alerts, the validation response form (VRF) supplied by *checkCIF* should be completed and included in the submitted CIF, preferably with the addition of appropriate explan­atory text in the deposited experimental section of the CIF.

The text and tables of an article may be previewed by sending the CIF (after completing the pre-check) to the *printCIF* service at **http://journals.iucr.org/services/cif/printcif.html** or by using *publCIF*. We strongly encourage authors to make use of this facility for checking the formatting of the supplementary materials.

### Categories of submission

*Section E* publishes two categories of articles. The requested cat­egory must be specified in the submitted CIF as _publ_requested_category, using one of the codes listed below.

(*a*) Short structural articles describe the determination of a single structure. The submitted CIF is validated using *checkCIF* and database checks are applied using the Inorganic Crystal Structure Database or the Cambridge Structural Database. The CIF, the checking results and associated files are then passed to a Co-editor for peer review. Once accepted, the article and associated graphics, together with the CIF and structure factors, will be accessible electronically from the **Crystallography Journals Online** service at **http://journals.iucr.org**. The category codes used to identify these articles are EI for inorganic, EM for metal–organic, and EO for organic compounds.

(*b*) *Addenda or Errata* are short articles describing additions to, comments on, or errata to existing *Section E* publications and are *not* intended for interim reports of work in progress. The text should not exceed 1000 words. *Addenda and Errata* are peer reviewed. The category code for these articles is AD.

### Method of submission

Full details of the submission procedure can be found at **http://journals.iucr.org/e/services/helpsubmit.html**. CIFs must be submitted *via* the web at **http://journals.iucr.org/e/services/submitbdy.html**. All submitted CIFs must have been pre-checked using the facilities described in §1.1. 

During the submission procedure, authors will be required to submit additional electronic files; these include the chemical scheme (see §2.8), crystallographic diagrams (see §3.5) and structure factors (see §3.8) or powder diffraction data (see §3.7). Authors will also be asked to agree to an open-access licence (see §1.11). In addition, they will also be asked to confirm that they can pay the open-access fee, or that they have a payment waiver (see §9).

On completion of the submission procedure, each article will be assigned an Editorial Office refcode. The Editorial Office refcode has two letters and four digits (e.g. hb3795), with the two letters identifying the assigned Co-editor; the refcode should be used in all subsequent communications with the Editorial Office and Co-editor.

### Handling of manuscripts

Each submitted CIF is automatically validated for completeness and data integrity, and checked for duplication. If incomplete or inadequate it will be returned to the contact author for correction. Some of the specific data standards are summarized in §4, while full details of the required data items and the data-validation criteria are available *via* the online CIF help page (see §8). For articles unable to meet these criteria, a completed validation response form (VRF) giving reasons for the failure must be included in the CIF. The Co-editor will assess the validity of the explanation as part of the review process.

The Co-editor is responsible for the review steps and future communications with the authors up to the acceptance stage. If, after review, no revisions are necessary, the article will be prepared for immediate electronic publication. If the review reveals that revision is required, the authors will be contacted directly and asked to revise the article. Further revisions may be requested before acceptance of the submission if unresolved issues remain. When the article is accepted by the Co-editor, the author will be asked to review the edited CIF and the HTML supplement, and approve these before proofs are prepared. The author will also be asked to pay the open-access fee or provide a waiver at this stage.

Failure to respond to a communication from either a Co-editor or the Editorial Office staff **within one month** will result in the automatic withdrawal of the article. All communications to authors will normally be sent electronically to the e-mail address provided in the CIF. Authors who anticipate or become aware of difficulties with their e-mail service should alert their Co-editor as soon as they are aware that there is a problem. If major revisions (*i.e.* revisions involving a complete new CIF) are made to the submission, the journal reserves the right to reset the date of receipt of the article to the date of resubmission. If a manuscript is not acceptable after two revisions it will not be considered further. An article that has been rejected must not be resubmitted to any IUCr journal unless the reasons given for the rejection have been fully addressed in the revised version.

Once an article is accepted, it is the responsibility of the Managing Editor to prepare the article for publication and to correspond with the authors and/or the Co-editor to resolve publication ambiguities or inadequacies. The Section Editors review all accepted articles and reserve the right to request or make appropriate changes to ensure conformity with *Section E* standards; in the unlikely event of significant changes being required at this stage, the authors will be contacted promptly.

### Revisions

After initial submission, any revised or new files should be uploaded *via* the web interface **only** in response to a specific request from a Co-editor; these files should be uploaded at the web address provided by the Co-editor.

### Author’s warranty

The submission of an article is taken as an implicit guarantee that the work is original, that it is the author(s) own work, that all authors are aware of and concur with the submission, that all workers involved in the study are listed as authors or given proper credit in the acknowledgements, that the manuscipt has not already been published (in any language or medium), and that it is not being considered and will not be offered elsewhere while under consideration for an IUCr journal. The inclusion of material in an informal publication, *e.g.* a preprint server or newsletter, does not preclude publication in an IUCr journal.

The co-authors of an article should be all those persons, and only those persons, who have made significant scientific contributions to the work reported, including the ideas and their execution, and who share responsibility and accountability for the results. Other contributions should be indicated in the acknowledgements. An administrative relationship to the investigation does not in itself qualify a person for co-authorship (but it may be appropriate to acknowledge major administrative assistance). Changes to the list of authors will normally require the agreement of the editor and all authors.

Important considerations related to publication have been given in the ethical guidelines published in *Acc. Chem. Res.* (2002), **35**, 74–76 and Graf *et al.* [*Int. J. Clin. Pract.* (2007), **61**(Suppl. 152), 1–26]. Authors are expected to comply with these guidelines.

### Submission of related structures

To allow handling of articles to be automated, all submissions should report single structures only. Series of single structure articles on closely related materials should not be merged. It is possible for submissions reporting related structures to be handled together and published as adjacent articles. Authors should select the same Co-editor for each article during submission. However, such studies may be more appropriately reported as a single article in *Acta Crystallographica Sections B* or *C*. Articles in a series should cross-reference each other. 

### Previously published structures

If a structure has been redetermined correctly and the result adds significantly to the information already in the public domain then the article can be considered for publication. Redeterminations that report a small improvement in precision or are merely carried out at a different temperature to previous studies will not normally be considered for publication. Unless they lead to significant insight, for example into reaction mechanisms or biological processes, determin­ations of the second enantiomer of a published first enantiomer of a structure will not be considered for publication. Redeterminations must cite the previous structure(s) and the *Abstract* must briefly state the changes and/or improvements attained.

### Languages of submission

The languages of publication are English, French, German and Russian. Submissions in English may use either British or American spelling, but must do so consistently throughout.

### Quality of writing

Articles must be clearly written and grammatically correct. If the Co-editor concludes that language problems would place an undue burden on the referee(s), the manuscript may be returned to the authors without review. Details of language-editing services can be found at **http://journals.iucr.org/services/languageservices.html.**

### Copyright

Authors will not be asked to transfer copyright to the IUCr, but will instead be asked to agree during article submission to an open-access licence. This licence is identical to the Creative Commons Attribution Licence.

### Author grievance procedure

An author who believes that an article has been unjustifiably treated by the Co-editor may appeal initially to the Section Editors for a new review and, finally, to the Editor-in-chief of IUCr Journals if the author is still aggrieved by the decision. The initial appeal must be made within 3 months of rejection of the article. The decision of the Editor-in-chief is final. Any resubmission to another Co-editor will be forwarded to the Section Editors.

## Publication requirements

The publication requirements for the text, tabular and graphical material are described in this section. Requirements for supplementary materials are given in §3, and the standards for numerical and codified data are summarized in §4. A list of all data items required for submission is available *via* the online CIF help page (see §8) where guidelines concerning the use of special characters (*e.g.* Greek letters and diacritical marks) and a set of Frequently Asked Questions are also given. Note that in the shorter article format the *Related literature* section (§2.3) replaces the *Comment* section; the *Comment* section, the *Experimental* text sections and the crystallographic diagrams now form part of the supplementary materials (§3). A chemical structural diagram must be included for metal-organic and organic compounds (§2.8).

### Title and authors

The *Title* should be short and informative; in many cases just the name of the compound studied will be perfectly adequate. However, the use of titles consisting of only the chemical formula is discouraged. Avoid redundant phrases such as ‘*Crystal structure of…*’. For articles describing powder, neutron or synchrotron diffraction studies, the title should typically consist of the name of the compound followed by ‘(powder study)’, ‘(neutron study)’ or ‘(synchrotron study)’, respectively. The full first name of each author is preferred. Note that the data items _publ_section_title_footnote and _publ_author_footnote are available, if required, for inserting footnotes to the title and to individual authors. If the article describes the redetermination of a previously reported structure, this should be indicated in the title. Part numbers for a series of related articles are not permitted in the title, but may be given as a title footnote.

### Abstract

The *Abstract* must be written in English, be informative, and should summarize the most important aspects of the study. It is expected to be no more than 250 words and should be capable of being understood on its own without access to the text or figures. It should not contain the crystal data or, usually, the space group. The systematic IUPAC name and the chemical formula should be given here, if they are not included in the *Title*. The *Abstract* should include mention of any crystallographically imposed symmetry or the presence of more than one molecule (or formula unit) in the asymmetric unit of the structure. Principal structural geometry results can be given here. Literature references should be avoided if possible; if required, they must be given in full, *e.g.* [Bond, Davies & Kirby (2001). *Acta Cryst*. E**57**, o1242–o1244].

### Related literature

All articles must include a *Related literature* section. Essential references (*e.g.* to the origin of the material studied, related structural studies, and to information supporting the reported structure) should be cited in this section, with appropriate very brief explanatory text, for example ‘The synthesis of the complex is described by Jones (2001). The anhydrous complex has essentially the same coordination geometry (Smith, 2003).’ In addition, all references included in the supplementary materials, but not elsewhere in the published article, should be given here. It is most useful for readers if the *Related literature* section is sub-divided, so that, instead of just "For related literature, see… ." it gives, for example, "For background to this class of compound, see… . For related structures, see… ." *etc*.

### Experimental data

Principal experimental data are extracted automatically from the CIF and are tabulated under the sub-headings *Crystal data*, *Data collection* and *Refinement*. Some numerical items may be formatted so that a standard number of decimal places is published. The full set of data in the CIF, as well as the content of the text sections _publ_section_exptl_prep, _exptl_special_details, _publ_section_exptl_refinement and _refine_special_details, will be provided in the supplementary materials. Authors should place a copy of their refinement instructions file(s), where available, in the _iucr_refine_instructions_details section and *publCIF* may be used for this purpose. This will facilitate the review of the manuscript, particularly when special refinement strategies, such as the use of restraints, have been employed Co-editors may request any additional experimental data or material they feel necessary to complete a full review of the article.

### Acknowledgements

Acknowledgement should be given for any assistance provided to the study (see §1.6). If the diffraction data collection was not carried out by one of the authors, or in the laboratory of one of the authors, details of who collected the data and where the data collection was carried out should be provided.

### References

References to published work must be cited in the format detailed in §7. If reference is made to unpublished work, prior consent must be first obtained from the authors of that work.

### Selected geometrical data

Full details of molecular dimensions, including those involving H atoms, should be supplied; only values that are of special interest should be flagged for publication by setting the _geom_..._flag value to yes. Bond lengths and angles should not normally be flagged for publication in organic structures. The data to be published in the article will be reviewed by the Co-editor and a Section Editor.

### Chemical scheme

A chemical structure diagram (typical examples are shown below) must be included for all but inorganic materials. Authors are required to submit such diagrams electronically in one of the formats listed in §5. The diagram should be complete, showing all species present in the structure, including counter-ions and solvent molecules in their correct proportions. Any relative or absolute stereochemistry, if this has been determined, must be shown and should be consistent with the crystallographic diagram (§3.5), the data reported in the *Abstract* (§2.2), *Related literature* (§2.3), *Comment* (§3.1) and *Title* (§2.1). Hydrogen bonding should not normally be indicated in the diagram. 
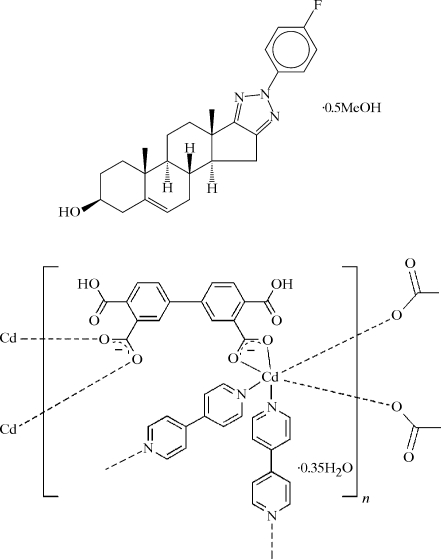


The chemical structure diagram should show only the compound whose structure is being reported. Chemical reaction schemes or schemes showing more than one compound should be supplied as supplementary figures and should have appropriate captions included in _publ_section_figure_captions (see §5.4).

Authors are encouraged to submit chemical connectivity (MOL, CML, CHM, SMI) files of reported structures with their articles. These files will be made available as part of the supplementary materials for each article and will be used to provide InChI (International Chemical Identifier) keys for the article, making the structures easier to find in the chemical literature.

### Powder diffraction data

Authors of powder diffraction articles should consult the notes provided at **http://journals.iucr.org/services/cif/powder.html**.

### Standard uncertainties

The standard uncertainty (abbreviated s.u. and replacing the traditional term estimated standard deviation) should be expressed as a number in parentheses following the numerical result and should be on the scale of the least significant digits of the result. The s.u. value should preferably be in the range 2–19. Note that s.u. values should not be appended to parameters which are fixed by symmetry, geometry or other constraints.

## Supplementary materials requirements

*Section E* provides extensive supplementary materials for each article. They include the supplementary data files (CIF, structure factors *etc.*), as well as (*a*) HTML and PDF representations of the deposited CIF and (*b*) the crystallographic diagrams. All supplementary materials will be available from **Crystallography Journals Online** (see §8.6). The text sections of the supplementary material (see §§3.1 and [Sec sec3.2]3.2) will be only lightly edited and checked for obvious factual errors, and authors may be asked to provide revised text if necessary.

### Comment

Where the submitted CIF includes a *Comment* section, this will be made available to readers in the HTML and PDF representations of the deposited CIF. Inclusion of a *Comment* section is strongly encouraged, especially to provide further information about the relevance of the *Related literature* and for discussion of the main structural results and their wider scientific context and significance, but the number of literature references should be small. The *Comment* section should also include a brief description of each crystallographic diagram (§5.1) provided by the authors.

### Experimental text sections

The content of the text sections _publ_section_exptl_prep, _exptl_special_details, _publ_section_exptl_refinement and _refine_special_details will be included in the HTML and PDF representations of the deposited CIF. The descriptive text item _publ_section_exptl_prep is the appropriate place to give information on the chemical and crystal preparation, and identification (*e.g.* on melting points and densities), the inclusion of which is encouraged. Additional details [*e.g.* lengthy synthetic descriptions and long lists of spectroscopic (NMR, IR *etc*.) data] supporting the crystallographic study should be placed in the _exptl_special_details section of the CIF. The text item _publ_section_exptl_refinement should be used to provide details of how H atoms were treated and report the ranges of *X*—H dimensions. Any unusual aspects of the data collection, space-group identification, data processing, structure determination and refinement (including any restraints used and the treatment of disorder) should also be given here. Routine material should be placed in _refine_special_details.

### Atomic sites

The _atom_site_ coordinate and atomic displacement param­eters (as *U^ij^*) must be supplied with standard uncertainty values (see §6.1 and §2.10). Any param­eter constraints and restraints applied during refinement should be documented (see also §4, _refine_ls_number_restraints). Note that only *U* or *U^i^^j^* values are acceptable for atomic displacement parameters. The atom numbering should follow some recognized scheme (see §6.1) and the atom list should be in some sensible (not random) order. H-atom labels should be directly related to the atom to which they are bonded.

### Geometrical data

Full details of molecular dimensions, including those involving H atoms, should be supplied. All submitted geometry data will be available to readers from **Crystallography Journals Online**. The inclusion of torsion angles in the CIF is encouraged, but should not normally include those involving H atoms; those in which three of the atoms are nearly collinear should be removed. 

### Crystallographic diagrams

Crystallographic diagrams will be available as part of the supplementary materials. Diagram requirements are given in §5. A labelled displacement ellipsoid diagram is normally required for each structure. Authors may supply additional figures *e*.*g*. packing diagrams. The use of colour is encouraged, but poor contrast (*e.g*. pale colours with a white background) should be avoided.

### Chemical reaction schemes

Chemical reaction schemes or schemes showing more than one compound will be available as part of the supplementary materials. They should be supplied as supplementary figures and should have appropriate captions included in _publ_section_figure_captions (see §5.4).

### Powder diffraction data

For articles that present the results of powder diffraction profile fitting or refinement (Rietveld) methods, the primary diffraction data, *i.e.* the numerical intensity of each measured point on the profile as a function of scattering angle, should be deposited. Articles reporting Rietveld refinements should include a supplementary figure showing the diffraction profile and the difference between the measured and calculated profiles.

### Structure factors

The reflection data *h*, *k*, *l*, *Y*_meas_, σ*Y*_meas_, *Y*_calc_ (where *Y* is *I*, *F* ^2^ or *F*) must be supplied as an electronic file in CIF format during the submission process. Note that in the case of structures determined from twinned crystals, the reflection data file used as input to the refinement program (*e.g.* the HKLF5 format file used by *SHELXL*) may be requested.

## Data requirements

A list of all data required for submission is available from the online CIF help page (see §8). If the submitted data are incomplete, inadequate or incorrect, the author will be informed promptly. Authors are required to pre-check each CIF (see §1.1) prior to submission. A more complete description of the data-validation checks applied to submitted CIFs is available from the online CIF help page (see §8).

Data-precision indicators will be published for all articles. Details of these can be found at **http://journals.iucr.org/services/cif/dataprecision.html**.

The most important data requirements are summarized below.

**_chemical_formula_moiety**

**_chemical_formula_sum**

The chemical formula must be consistent with the atomic content specified by the _atom_site_ information, and match the _chemical_formula_weight. If atoms are missing from the atomic model, the moiety and sum formulae should state the assumed overall formula.

**_symmetry_space_group_name_H-M**

The space group must encompass the highest symmetry permitted by the diffraction intensities, and be consistent with the _cell_length_ and _cell_angle_ values.

**_cell_formula_units_Z**

The number of formula units in the unit cell must comply with that expected from the chemical formula, the space group and the _atom_site_ data.

**_exptl_crystal_colour**

The crystal colour should comply with the codes listed in the online author help page (see §8).

**_exptl_crystal_size_max**

Authors are encouraged to use crystals no larger than the incident X-ray beam diameter, particularly when heavy or strongly absorbing elements are present in the material. For best results, the crystal should be uniformly bathed in the X-ray beam. The size of the beam at the crystal is normally determined by *inter alia* the nature of the X-ray source and the beam optics. Note that with fine-focus sealed X-ray tubes, the use of a collimator larger than the filament diameter does not automatically increase the size of the uniform part of the incident beam.

**_exptl_absorpt_correction_type**

Permitted absorption-type codes are listed in the online CIF help page (see §8). A type code must be accompanied by a reference to the method or the software used; this should be given in the field _exptl_absorpt_process_details. The need for absorption corrections, and the appropriate type of correction, is dependent on the μ value, _exptl_absorpt_coefficient_mu, and the crystal size values, _exptl_crystal_size_min, _mid and _max. If *x* is the medial size _mid, the product μ*x* provides an indication of the type of correction needed. Analytical or numerical corrections may be beneficial if μ*x* exceeds 1.0 and are strongly recommended if μ*x* is above 3.0. However, corrections based on analyses of equivalent and redundant reflections (multi-scan methods) are acceptable. Corrections are usually unnecessary if μ*x* is below 0.1. Refined absorption methods are discouraged except in special circumstances. The experimentally determined transmission-factor limits _exptl_absorpt_correction_T_min and _max should be consistent with those expected for the crystal shape and size, and μ.

Whenever a multi-scan type absorption correction is being employed (*e.g.* by using *SADABS*), authors are also encouraged to measure a multiplicity of observations (measurement of symmetry equivalents or the same reflection at different crystal orientations) of at least 4. The algorithms used in such programs work best and produce the highest quality data only when the multiplicity of observations or coverage of the full sphere of reflections is high.

**_diffrn_reflns_number**

**_diffrn_reflns_av_R_equivalent**

**_diffrn_reflns_limit_h_min**

**_diffrn_reflns_limit_h_max**

**_diffrn_reflns_limit_k_min**

**_diffrn_reflns_limit_k_max**

**_diffrn_reflns_limit_l_min**

**_diffrn_reflns_limit_l_max**

These items should refer to the complete set of measured data before any merging of symmetry-equivalent reflections, and not only to the unique set of data.

**_reflns_number_total**

The number of symmetry-independent reflections excludes the systematically extinct intensities. Authors are encouraged to use **all** symmetry-independent reflections in the refinement of the structure parameters.

**_reflns_threshold_expression**

This threshold, which is based on multiples of σ*I*, σ*F* ^2^ or σ*F*, serves to identify the significantly intense reflections, the number of which is given by _reflns_number_gt. These reflections are used in the calculation of _refine_ls_R_factor_gt. The multiplier in the threshold expression should be as small as possible, typically 2 or less.

**_diffrn_reflns_theta_max**

The θ_max_ of measured reflections should be such that sin θ_max_/λ exceeds 0.6 Å^−1^ (*i.e*. θ_max_ > 25° for Mo *K*α; θ_max_ > 67° for Cu *K*α). It is expected that all possible unique reflections out to at least the above specified minimum θ limits are measured. This provides the minimum number of reflections recommended for an average structural study. If intensities are consistently weak at the recommended θ_max_, low-temperature measurements may be needed unless a study at a specific temperature (or pressure) is being reported.

**_diffrn_measured_fraction_theta_max**

This is the fraction of unique (symmetry-independent) reflections measured out to _diffrn_reflns_theta_max. Ideally, this should be as close to 1.0  as possible.

**_diffrn_reflns_theta_full**

When _diffrn_measured_fraction_theta_max is less than 1.0 because of some missing high-angle reflections, θ_full_ is the diffrac­tometer angle at which the measured reflection count is close to complete. The fraction of unique reflections measured out to this angle is given by _diffrn_measured_fraction_theta_full.

**_diffrn_reflns_av_R_equivalents**

Sufficient symmetry-equivalent reflections must be measured to provide a good estimate of the intensity reproducibility. This is particularly important when absorption corrections are applied (this value is calculated *after* the corrections are applied to the intensities). See also _exptl_absorpt_correction_type.

**_refine_ls_R_factor_gt**

Note that this value is **not** intended as a reliable gauge of structure precision, which is better determined from the standard uncertainties of the parameters (these depend on the number and reliability of the measured structure factors used in the refinement process).

**_refine_ls_number_reflns**

The number of reflections used in the refinement should be as large as possible, and should, if possible, be greater than the number of refined parameters _refine_ls_number_parameters by at least a factor of 10 if the structure is centrosymmetric, or by a factor of 8 if it is not. Omission of outlier reflections should be avoided unless there is good reason and, in such cases, details of the omitted reflections and the reasons for doing so should be included in the _publ_section_exptl_refinement section.

**_refine_ls_number_parameters**

This is the number of coordinate, atomic displacement, scale, occupancy, restraint, extinction and other par­ameters refined independently in the least-squares process. It is possible, and sometimes desirable, to reduce this number by the appropriate application of geometric constraints.

**_refine_ls_number_restraints**

This gives the number of applied restraints. Concise details of what these restraints were, including any target values applied and the effective standard deviation of the restraint, should be included in the _refine_special_details section of the CIF.

**_refine_ls_hydrogen_treatment**

The codes which identify the treatment of H-atom par­ameters are listed in the online CIF help page (see §8). Detailed text about the treatment of H-atom sites should be placed in _publ_section_exptl_refinement. Authors should note the advice on H-atom treatment given in the *SHELXL*97 manual, §4.6: ‘*For most purposes it is preferable to calculate the hydrogen positions according to well-established geometrical criteria and then adopt a refinement procedure which ensures that a sensible geometry is retained*’. Authors should note that H-atom sites which have been fixed or constrained by geometry (*e.g.* riding) will not have s.u. values associated with them.

**_refine_ls_weighting_scheme**

Weighting schemes for refinements should be based on the standard uncertainties in the measured reflection data.

**_refine_ls_shift/su_max**

This is the largest ratio of the parameter shift to standard uncertainty after the final round of refinement and is typically within ±0.01 if sufficient least-squares refinement cycles have been employed. A value above ±0.05 is considered unusual and values beyond ±0.1 are a sign of incomplete refinement, unaccounted-for disorder or high correlation between parameters that should be constrained. Authors should explain the reasons for a high value in _publ_section_exptl_refinement.

**_refine_diff_density_min**

**_refine_diff_density_max**

These values are expected to be small, especially for light-atom structures. If their magnitudes are such that a validation alert is generated, the label and the distance of the closest atom site should be reported in _publ_section_exptl_refinement.

**_geom_**

All geometry values must originate from the submitted _atom_site_fract_ values. Only geometry values of significance to the structure will be published. These must be identified with a _geom_..._flag value of yes in the submitted CIF. Note that dimensions involving H-atom sites which have been fixed or constrained by geometry will not have s.u. values associated with them. Details of all bond lengths and angles involving H atoms must be included in the CIF, even if they have been constrained.

**_atom_site_**

Atomic coordinates for molecular structures should be supplied as connected sets. Whenever structure geometry permits, it is normally expected that the set of connected coordinates which specify the asymmetric unit will lie within the basic unit cell. Values of _atom_site_occupancy should be 1.0 except for disordered or non-stoichiometric atom sites. Atom sites constrained to model disorder must be indicated by _atom_site_disorder_group. The overall packing in the structure will be checked for significant vacant regions (*i.e*. voids) indicating omitted solvent molecules. Note that s.u. values should not be appended to parameters which are fixed by symmetry, geometry or other constraints. In systems with hydrogen-bonded networks, it is expected that the asymmetric unit will be chosen so that the minimum number of symmetry operators is required to specify the hydrogen-bond network.

**_atom_site_aniso_U_**

Checks will be made for non-positive-definite anisotropic atomic displacement parameters. The ratio of maximum to minimum eigen­values should not, except in special circumstances (*e.g*. disorder), exceed 5.

**_refine_ls_abs_structure_details**

This item should describe the method applied, with a literature citation if necessary, and the number of Friedel pairs used in the determination of the absolute structure parameter (*e.g*. _refine_ls_abs_structure_Flack). If the structure is non-centrosymmetric, an absolute structure parameter is expected. The reliability of this parameter increases with the number of Friedel-related intensities, and the use of a large fraction of the complete set of Friedel pairs in the refinement is strongly recommended. Even if the atomic composition and the choice of X-ray wavelength mean that the *f*′′ terms of the atomic scattering factor expressions are very small, and the value of the absolute structure parameter is inconclusive because of its large s.u. value, it is not necessary to merge Friedel-pair reflections. If authors do merge Friedel-pair reflections before final refinement, they should mention that fact in the _publ_section_exptl_refinement section and not report a value for the Flack parameter in the CIF. Authors are strongly advised to consult articles by Flack & Bernardinelli [*Acta Cryst.* (1999), A**55**, 908–915; *J. Appl. Cryst.* (2000), **33**, 1143–1148] and by Flack, Sadki, Thompson & Watkin [*Acta Cryst.* (2011), A**67**, 21–34]. For pertinent comments on the determination of absolute structure authors are also referred to the articles by Jones [*Acta Cryst*. (1986), A**42**, 57] and Hooft *et al.* [*J. Appl. Cryst.* (2008), **41**, 96–103; *J. Appl. Cryst.* (2010), **43**, 665–668].

## Diagram requirements

A set of guidelines for preparing figures is available from **http://journals.iucr.org/j/services/help/artwork/guide.html**. The chemical structure diagram (see §2.8 for a typical example) and supplementary crystallographic diagrams (see §3.5) should be prepared in HPGL, PostScript, encapsulated PostScript or TIFF format. The resolution of bitmap graphics should be a minimum of 300 d.p.i. 

### Supplementary crystallographic diagrams

For molecular compounds, a clear, well-presented ellipsoid plot is normally required for each independent species to show the stereochemistry and any unusual atomic displacements or disorder. For polymeric materials the displacement ellipsoid plot should show at least the chemically unique fragment; for other structures, a packing or polyhedron diagram is required. All non-H unique atom sites should be identified with labels consistent with those for the supplied atomic coordinates. Distances and angles should not be shown in the crystallographic diagram. The orientation of crystallographic figures and chemical structural diagrams should correspond as closely as possible.

Diagrams should be briefly described in the *Comment* section for the benefit of readers.

### Submission

Diagrams should be submitted electronically *via* the web submission interface. All diagrams must be submitted in this way.

### Lettering and symbols

Atom site labels in supplementary crystallographic diagrams should not contain parentheses and should match labels used in the atom site lists and text. The labels should not overlap or touch ellipsoids or bonds. Descriptive matter should be placed in the caption. Packing diagrams should show the unit-cell outline, with the cell-axis directions (labelled *a*, *b*, *c*) and the cell origin (labelled *O*) marked thereon or given in a legend at the side.

### Numbering and figure captions

A list of the figure captions should be included in _publ_section_figure_captions. Captions of labelled displacement ellipsoid plots must state the probability limit used. If H atoms are shown by small spheres of an arbitrary size, this need not be stated in the caption.

### Enhanced figures

An online tool for authors to prepare standard and corresponding three-dimensional interactive structural diagrams is available from **http://submission.iucr.org/jtkt**.

## Nomenclature

### Crystallographic nomenclature

Authors should follow the general recommendations produced by the IUCr Commision on Crystallographic Nomenclature (see reports at http://www.iucr.org/iucr/commissions/cnom.html).

Atom sites not related by space-group symmetry should be identified by unique labels composed of a number appended to the IUPAC chemical symbol (*e.g.* C5, C7 *etc*.). Label numbers should not be placed in parentheses. **Chemical and crystallographic numbering should be in agreement wherever possible.** Crystallographically equivalent atoms in different asymmetric units should be identified in the text with lower-case Roman numeral superscripts appended to the original atom labels and the symmetry operators defined [*e.g*. C5^i^; symmetry code: (i) −*x* + 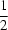, *y* − 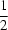, −*z* + 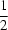]. In diagrams, Roman numeral superscripts are preferred, but symmetry-related atoms may be marked by additional symbols or letters, with these defined in the caption. Atom labels should be as concise as possible and not contain superfluous characters, *e.g.* leading zeroes are unnecessary so that C01, C02 should appear as C1, C2, *etc*. H-atom numbers should correspond with that of their parent atom label, *e.g.* a phenyl H on atom C1 should appear as H1, not H1A, and methylene H atoms on C1 should appear as H1A and H1B or H11 and H12, unless this leads to naming ambiguities. Chemically ambiguous labels such as HO1a (signifying an H atom attached to an O atom) should be avoided. If there are supplementary figures with packing plots in an article, the atom labels in these plots should correspond with the asymmetric-unit coordinates. Symmetry-related atoms can be marked by suffixes to the atom labels; the symmetry operations to which these refer should be defined in the figure caption.

Space groups should be designated by the Hermann–Mauguin symbols. In triclinic systems, the reduced cell must be used, and for other crystal systems, standard cell settings, as listed in Volume A of *International Tables for Crystallography*, should be used unless objective reasons to the contrary are stated. A list of equivalent positions should also be supplied. Hermann–Mauguin symbols should be used for designating point groups and crystallographically-imposed molecular symmetry. If there is a choice of origin, this should be stated in _publ_section_exptl_refinement.

### Nomenclature of chemical compounds

Names of chemical compounds and minerals should conform to the nomenclature rules of the International Union of Pure and Applied Chemistry (IUPAC), the International Union of Biochemistry and Molecular Biology (IUBMB), the International Mineralogical Association (IMA) and other appropriate bodies. Any accepted trivial or non-systematic name may be retained, but the corresponding systematic (IUPAC) name should also be given. For crystal structures containing chiral molecules, authors should make it clear whether the crystal structure is a racemate or enantiopure, and if enantiopure whether or not the assignment of the absolute configuration is justified. A corresponding CIF code should be entered into the data item _chemical_absolute_configuration. The title, compound name, chemical diagrams, atomic coordinates and space group must correspond to the enantio-composition and the selected configuration. It is also most helpful to indicate the crystallographic and non-crystallographic symmetry of each molecule in the asymmetric unit. If the absolute configuration has not been determined from the diffraction data or assigned with reference to a compound of known absolute configuration, the title and chemical scheme should not imply a known absolute structure.

Authors may find nomenclature programs such as AutoNom (http://www.mimas.ac.uk/crossfire/autonom.html) and ACDLABS (http://www.acdlabs.com/products/name_lab) to be useful resources when naming compounds.

### Units

The International System of Units (SI) is used except that the ångström (symbol Å, defined as 10^–10^ m) is generally preferred to the nanometre (nm) or picometre (pm) as the appropriate unit of length. Recommended prefixes of decimal multiples should be used rather than ‘×10^*n*^’.

## References

All references cited in the article and in the supplementary materials should be given in the reference list. An extensive list of references is inappropriate for manuscripts submitted to this journal, which is intended for short structural reports. Manuscripts giving a substantive discussion of a crystal structure and including many references are more appropriately submitted to *Acta Crystallographica Section C*.

References to published work must be indicated by giving the authors’ names followed immediately by the year of publication, *e.g.* Neder & Schulz (1999) or (Neder & Schulz, 1999). Where there are three or more authors the reference in the text should be indicated in the form Smith *et al.* (1989) or (Smith *et al.*, 1989) *etc*., unless more authors need to be given to make the citation unambiguous. Thus, for the following two references:

Smith, A. B., Jones, C. D., Wolf, E. F., Baird, M. N. & Hunt, G. H. (1994). *Organometallics*, **13**, 123–128.

Smith, A. B., Jones, C. D., Lyle, R. S., Hunt, G. H. & Baird, M. N. (1994). *Chem. Rev.* 
            **94**, 345–380.

the reference in the text should be distinguished as follows: Smith, Jones, Wolf *et al.* (1994) and Smith, Jones, Lyle *et al.* (1994).

In the reference list (_publ_section_references), the ordering of references should be: 

(1) First, alphabetically by surname of first author, then second author *etc*.

(2) Second, numerically by year for the same set of authors.

For references with identical lists of authors and the same year of publication, the *a*,* b* 
            *etc*. notation is used, *e.g.*
         

Drew, M. G., Hall, R. S. & Long, F. I. (2004*a*). *Organometallics*, **23**, 456–459.

Drew, M. G., Hall, R. S. & Long, F. I. (2004*b*). *J. Med. Chem.* 
            **47**, 579–586.

Lehmler, H. J., Robertson, L. W. & Parkin, S. (2005). *Acta Cryst*. E**61**, o3025–o3026.

Lehmler, H. J., Robertson, L. W., Parkin, S. & Brock, C. P. (2002). *Acta Cryst.* E**61**, o123–o124.

Entries for journals should be abbreviated in the style of *Chemical Abstracts* (the abbreviations *Acta Cryst., J. Appl. Cryst.* and *J. Synchrotron Rad.* are exceptions), and the reference list should conform with the following style:

Albada, G. A. van, Mutikainen, I., Turpeinen, U. & Reedijk, J. (2001). *Acta Cryst*. E**57**, m421–m423.

Back, S., Rheinwald, G., del Rio, I., van Koten, G. & Lang, H. (2001). *Acta Cryst*. E**57**, m444–m445.

Bond, A. D. (2003). *Acta Cryst*. E**59**, o1992–o1993.

Bond, A. D., Clayden, J. & Wheatley, A. E. H. (2001). *Acta Cryst*. E**57**, o292–o294.

Bond, A. D. & Davies, J. E. (2002). *Acta Cryst*. E**58**, o5–o7.

Bond, A. D., Davies, J. E. & Kirby, A. J. (2001). *Acta Cryst*. E**57**, o1242–o1244.

Bruker (1998). *SMART* and *SAINT*. Bruker AXS Inc., Madison, Wisconsin, USA.

Bürgi, H.-B. (1989). *Acta Cryst*. B**45**, 383–390.

Filgueiras, C. A. L., Horn, A. Jr, Howie, R. A., Skakle, J. M. S. & Wardell, J. L. (2001). *Acta Cryst*. E**57**, o338–o340.

Hervieu, M. & Raveau, B. (1983*a*). *Chem. Scr*. **22**, 117–122.

Hervieu, M. & Raveau, B. (1983*b*). *Chem. Scr*. **22**, 123–128.

Hummel, W., Hauser, J. & Bürgi, H.-B. (2012). In preparation.

International Union of Crystallography (2001). (IUCr) Structure Reports Online, http://journals.iucr.org/e/journalhomepage.html

Jones, P. T. (2012). Personal communication.

Kiser, P. D., Lodowski, D. T. & Palczewski, K. (2012). *Acta Cryst*. F**68**, doi:10.1107/S1744309107020295.

Kooijman, H. & Spek, A. L. (2000). *Acta Cryst*. C**56**, e295–e296.

McCrone, W. C. (1965). *Physics and Chemistry of the Organic Solid State*, Vol. 2, edited by D. Fox, M. M. Labes & A. Weissberger, pp. 725–767. New York: Interscience.

Meyer, M. B., Zella, L. A., Nerenz, R. D. & Pike, J. W. (2008). *J. Biol. Chem*. doi:10.1074/jbc.M703475200.

Nonius (1997). *KappaCCD Server Software*. Nonius BV, Delft, The Netherlands.

Perkins, P. (2002). PhD thesis, University of London, England.

Robinson, P. D. (2012). *Acta Cryst*. E**68**. In the press. [Article reference sj3995].

Sheldrick, G. M. (2008). *Acta Cryst*. A**64**, 112–122.

Smith, J. V. (1988). *Chem. Rev*. **88**, 149–182.

Smith, J. (2000). Private communication (refcode: PYMTLI01). CCDC, Cambridge, England.

Smith, J. V. & Bennett, J. M. (1981). *Am. Mineral*. **66**, 777–788.

Vogel, A. (1978). *Textbook of Practical Organic Chemistry*, 4th ed. London: Longman.

Note that all authors and **inclusive** page numbers must be given, except when the cited article has only one page, and that all computer programs given in the relevant _computing_..._ entries must be referenced and included in the reference list. Note that version numbers should not be given for computer programs. Identification of individual structures in the article by use of database reference (identification) codes should be accompanied by a full citation of the original literature in the reference list. The CIF editor, *publCIF* (see §1.1), allows the consistency of references and citations to be checked.

## Author information and services

Full details of guidelines for the preparation and editing of a CIF, the data items required therein, standard data codes and keywords, CIF templates, example CIFs, and data-validation criteria and procedures can be found *via* the online author help page at **http://journals.iucr.org/e/services/cifinfo.html**.

### Author tools

A number of tools are available to help with the preparation of articles.

The *checkCIF/PLATON* service at **http://journals.iucr.org/services/cif/checking/checkfull.html** allows CIFs and structure factors to be checked.

CIFs can be edited using *publCIF*, available from **http://publcif.iucr.org**, or *enCIFer*, available from **http://www.ccdc.cam.ac.uk/products/encifer/index.html**.

A toolkit for preparing enhanced figures is available at **http://submission.iucr.org/jtkt**.

A preview of an article may be printed using the *printCIF* service at **http://journals.iucr.org/services/cif/printcif.html**.

### Example of a submission and its published version

An example CIF submission can be found at **http://journals.iucr.org/services/cif/examples.html**; this article has been published in the March 2007 issue of the journal [Clegg & Martin (2007). *Acta Cryst.* E**63**, m856] to show the features of the short-format style articles.

### Electronic status information

Authors may obtain information about the current status of their article at **http://journals.iucr.org/services/status.html**.

### Proofs

Proofs will be provided electronically as PDF files, and the contact author will be notified by e-mail when the proofs are ready for downloading. Any proof corrections must be returned within two days. Proofs will have been checked by the Section Editors, who reserve the right to make minor changes for consistency and conformity to *Section E* standards; any remaining problems will be referred back to authors.

### Reprints

After publication, the contact author will be able to download the electronic reprint of the published article, free of charge.

### Crystallography Journals Online

All IUCr journals are available on the web *via* **Crystallography Journals Online;** **http://journals.iucr.org/**. 

## Open access

*Acta Crystallographica Section E* is an open-access journal. The costs of peer review, of journal production, and of online hosting and archiving will be met by charging an open-access fee to authors.

Authors will be asked to pay an open-access fee upon acceptance of their article for publication. Authors from developing countries may apply for their open-access fees to be waived. Waiver requests must be made before submission of an article to the journal.

Authors whose institution has a full subscription to *Acta Crystallographica Section C* are eligible for a discount on the open-access fee. In addition, discounts are available for advance payment of open-access fees.

Full details of the open-access arrangements for Section E are available at **http://journals.iucr.org/services/oa/openaccess_e.html**.

